# MacroH2A1 downregulation enhances the stem-like properties of bladder cancer cells by transactivation of Lin28B

**DOI:** 10.1038/onc.2015.187

**Published:** 2015-06-01

**Authors:** S-J Park, J W Shim, H S Park, D-Y Eum, M-T Park, J Mi Yi, S H Choi, S D Kim, T G Son, W Lu, N D Kim, K Yang, K Heo

**Affiliations:** 1 Research Center, Dongnam Institute of Radiological and Medical Science (DIRAMS), Busan, Republic of Korea; 2 Department of Pharmacy, Pusan National University, Busan, Republic of Korea; 3 The Eli and Edythe Broad Center for Regenerative Medicine and Stem Cell Research, Department of Biochemistry and Molecular Biology, University of Southern California, Los Angeles, CA, USA; 4 Department of Radiation Oncology, Dongnam Institute of Radiological & Medical Sciences, Busan, Republic of Korea; 5 Department of Radiation Oncology, Korea Institute of Radiological & Medical Sciences, Seoul, Republic of Korea

## Abstract

The histone variant, macroH2A1, has an important role in embryonic stem cell differentiation and tumor progression in various types of tumors. However, the regulatory roles of macroH2A1 on bladder cancer progression have not been fully elucidated. Here, we show that macroH2A1 knockdown promotes stem-like properties of bladder cancer cells. The knockdown of macroH2A1 in bladder cancer cells increased tumorigenicity, radioresistance, degeneration of reactive oxygen species, increased sphere formation capability and an increase in the proportion of side populations. We found that macroH2A1 is required for the suppression of Lin28B identified as a novel downstream target of macroH2A1 in bladder cancer. Loss of macroH2A1 expression significantly correlated with the elevated levels of Lin28B expression and subsequently inhibited the mature let-7 microRNA expression. Furthermore, the stable overexpression of Lin28B enhances the several phenotypes, including tumorigenicity and sphere-forming ability, which are induced by macroH2A1 depletion. Importantly, Lin28B expression was regulated by macroH2A1-mediated reciprocal binding of p300 and EZH2/SUV39H1. Our results suggest that Lin28B/let-7 pathway is tightly regulated by macroH2A1 and its cofactors, and have a pivotal role in the bladder tumor progression and the regulation of stem-like characteristics of bladder cancer cells.

## Introduction

Histone variants have key roles in regulating chromatin structure and dynamics. The histone variant macroH2A (mH2A) contains an N-terminal domain that is homologous to conventional H2A and a C-terminal domain of approximately 30 kDa called the macrodomain. In mammalian cells, there have been three different forms of mH2A identified, mH2A1.1, mH2A1.2 and mH2A2. These three isoforms were initially reported to be involved in the inactive X chromosome of female mammals.^1^ Several additional studies have shown that mH2A contributes to the transcriptional repression of its target genes through interfering with the binding of transcription and nucleosome remodeling factors.^2, 3^ In addition, recent studies have suggested that mH2A can act as a tumor suppressor in many different human cancers. It has been shown that the expression of mH2A is significantly downregulated in many types of tumor tissues such as bladder, lung and breast, and that the loss of mH2A is able to promote cancer progression along with the metastatic potential of melanoma and bladder cancer cells.^4, 5, 6, 7^ Alternatively, several studies have suggested that mH2A also has a critical role in embryonic and induced pluripotent stem cell regulation and differentiation.^8, 9, 10, 11^

The RNA binding protein Lin28 and its homologue, Lin28B, are proteins that contain both a cold shock domain and a zinc finger domain.^12, 13^ These proteins bind to the terminal loops of let-7 microRNA (miRNA) precursors and suppress the production of mature let-7 miRNAs which act as tumor suppressor miRNAs.^14, 15^ Lin28 is highly expressed in mammalian embryonic stem cells,^16^ and it generates induced pluripotent stem cells from human somatic fibroblasts in association with OCT4, SOX2 and NANOG.^17^ Interestingly, a recent study has suggested that Lin28B is an oncofetal cancer stem-like cell marker for the recurrence of hepatocellular carcinoma.^18^ Moreover, several reports have suggested that Lin28 and Lin28B increase the expression of oncogenic regulators such as Myc, Ras and HMGA2 during cancer progression through let-7 miRNA repression.^19^

The small fraction of cells within a tumor that possess properties that are found normally in stem cells, such as self-renewal and the ability to differentiate into progeny cells are called cancer stem-like cells. Several studies suggests that cancer stem-like cells are responsible for cancer initiation, progression and metastasis, and are correlated with higher chemo- and radio-resistance.^20, 21^ Although a large body of evidence suggests a role for mH2A in stem cell regulation, the regulatory mechanism of mH2A in cancer stem-like cell activation remains unclear. In this study, we investigated the roles of mH2A1 specifically in stem-like properties of bladder cancer cells. Our results revealed that depletion of mH2A1 enhances stem-like properties such as tumorigenicity, migration, sphere formation and radioresistance. Lin28B, which is a novel downstream target of mH2A1, acts as a key regulator of these effects by antagonizing let-7 miRNAs. Besides, the selective bindings of p300 or EZH2/SUV39H1 modulated by mH2A1 participate in the regulatory mechanism of Lin28B expression. These findings provide new insight into the novel mechanism for bladder cancer progression, which is mediated by mH2A1/Lin28B/let-7 pathway.

## Results

### Knockdown of mH2A1 enhances the stem-like properties of bladder cancer cells

**Figure 1:**
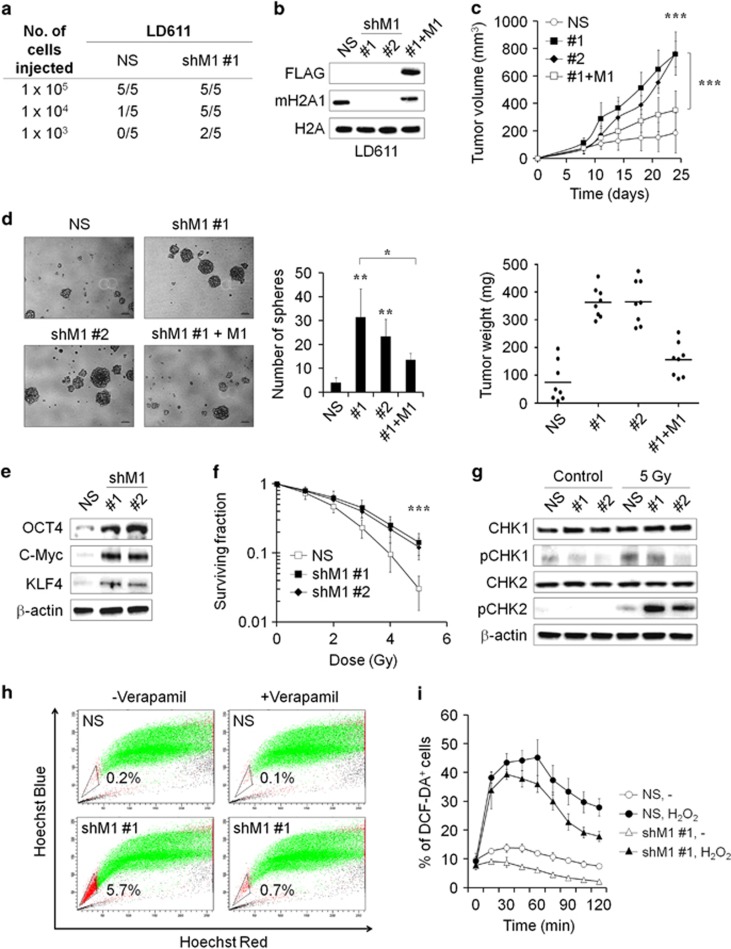
mH2A1 depletion enhances the stem-like properties of bladder cancer cells. (**a**) Non-silencing control shRNA-expressing (NS) or sh-mH2A1-expressing (shM1) LD611 cells were injected subcutaneously into SCID mice (*n*=5). Tumor formation was monitored for 30 days post injection. (**b**) The efficiency of mH2A1 knockdown or re-expression in LD611 cells was evaluated with western blot analysis. (**c**) NS or shM1 or mH2A1 re-expressed LD611 cells were injected subcutaneously into nude mice (*n*=8). The average tumor size was measured at different times (top), and the tumor weight was measured at the endpoint (bottom). (**d** and **e**) NS or shM1 LD611 cells were incubated with DMEM/F12 supplemented with B27, N2, epidermal growth factor and basic fibroblast growth factor. Spheres were counted and visualized after 1 week (**d**). Scale bars: 100 μm. The expression of pluripotency factors was determined by western blot analysis (**e**). Representative data (**f**) NS or shM1 LD611 cells were left untreated or were treated with 1–5 Gy of IR, and cell survival rates were determined using the clonogenic assay. (**g**) IR (5 Gy)-treated or -untreated NS or shM1 LD611 cells were lysed after 15 min of incubation. Checkpoint response proteins (total or phospho-CHK1 and CHK2) were evaluated by western blot analysis. (**h**) NS or shM1 LD611 cells were stained with 5 μg/ml of Hoechst 33342 alone or in the presence of verapamil (150 μM) and, side population cells were analyzed by fluorescence-activated cell sorting. (**i**) NS or shM1 LD611 cells were treated with H_2_O_2_ (400 μM) to induce oxidative stress. ROS concentrations were determined by 2′,7′-dichlorofluorescein diacetate assay staining. *P*-values were calculated using the Student's *t*-test. *P<0.05, **P<0.01, ***P<0.001.

mH2A1 knockdown significantly enhances bladder cancer cell proliferation and invasion. Furthermore, although mH2A1 is highly expressed in normal bladder tissues, it gradually declines with increases in malignant potential of the cancer.^7^ As these results suggested that the lack of mH2A1 is associated with the occurrence and progression of bladder cancer, we investigated whether the level of mH2A1 expression is associated with acquisition of bladder cancer stem cell-like properties. We accordingly generated mH2A1-depleted bladder cancer cell lines using two different shRNAs (#1 and #2) against mH2A1 (Supplementary Figure S1a; Figure 1b). Consistent with previous studies, cell proliferation increased in all mH2A1-depleted cell lines tested (Supplementary Figures S1b and c). Moreover, the depletion of mH2A1 enhanced the ability of bladder cancer cells to migrate and metastasize (Supplementary Figure S2a). Notably, the loss of mH2A1 induced the expression of epithelial–mesenchymal transition markers (Supplementary Figure S2b).

As tumorigenic capacity is a major characteristic of cancer stem-like cells, LD611 cells transfected with non-silencing control shRNA (NS) or sh-mH2A1 (shM1) were injected subcutaneously into severe combined immunodeficiency (SCID) mice. Thirty days after injection, tumors formed in SCID mice injected with 1 × 10^3^ shM1 cells (*n*=2/5). In contrast, a tumor mass appeared in only one of the five SCID mice injected with 1 × 10^4^ NS cells (Figure 1a; Supplementary Figure S3a). Similarly, nude mice transplanted with mH2A1-depleted 5367 cells also had larger sized tumors than those injected with control cells (Supplementary Figure S3b). mH2A1-depleted LD611 cells were re-transfected with mH2A1 overexpression vector (#1+M1) to further confirm the role of mH2A1 in tumor progression. Western blotting results confirmed that mH2A1 expression was efficiently recovered by the stable transfection of mH2A1 overexpression plasmid in the mH2A1-depleted cells (Figure 1b). Thereafter, nude mice were transplanted with mH2A1 knockdown (#1 and #2) or re-expressed (#1+M1) LD611 cells. The results revealed that the depletion of mH2A1 in LD611 cells dramatically increased the rate of tumor growth, while re-expression of mH2A1 attenuated tumor growth induced by mH2A1 depletion (Figure 1c).

To elucidate the significance of mH2A1 on stem-like properties, we next determined the role of mH2A1 in tumor sphere formation. mH2A1 depletion significantly induced sphere formation in LD611 cells, and this induction was partially restored by mH2A1 re-expression (Figure 1d). In addition, the expression levels of reprogramming factors (OCT4, C-Myc and Klf4) were increased in the spheres from the mH2A1-depleted LD611 cells (Figure 1e). Next, we investigated the survival rates of NS- or shM1-expressing LD611 cells after irradiation (IR) with 1–5 Gy of γ-rays. Our results revealed that the mH2A1-depleted LD611 cells were more resistant to radiation than the control cells (Figure 1f). As activation of DNA damage checkpoint responses promotes radioresistance in glioma stem cells and nasopharyngeal carcinoma cells,^22, 23^ we compared early DNA checkpoint responses in NS and shM1 LD611 cells. The results indicated that CHK2 phosphorylation was higher in shM1 cells than NS cells after treatment with 5 Gy IR, whereas the level of CHK1 phosphorylation was unchanged by IR in NS and shM1 LD611 cells (Figure 1g). A repair mechanism mediated by CHK2 phosphorylation might be responsible for the enhancement of radioresistance in shM1 LD611 cells.

Side population (SP) fraction has stem cell-like properties in diverse tumor tissues including bladder cancer.^24, 25, 26^ The NS cell population contained a small proportion of SP cells (0.2%), while the percentage of SP cells in the shM1 cells increased up to 5.7% (Figure 1h). The SPs disappeared after treatment with the calcium channel blocker, verapamil, thus resulting in the inhibition of Hoechst33342 uptake. Cancer stem-like cells reportedly generate lower levels of reactive oxygen species (ROS) than non-tumorigenic progeny.^27^ Therefore, we compared ROS levels in NS and shM1 LD611 cells. The results showed that shM1 cells contained significantly lower concentrations of ROS than NS cells. Furthermore, H_2_O_2_-induced ROS generation was abolished more rapidly in shM1 cells than NS cells (Figure 1i). The results collectively indicated that mH2A1 is one of the key regulators of stem-like properties of bladder cancer cells.

### Lin28B is a novel downstream target for mH2A1

**Figure 2:**
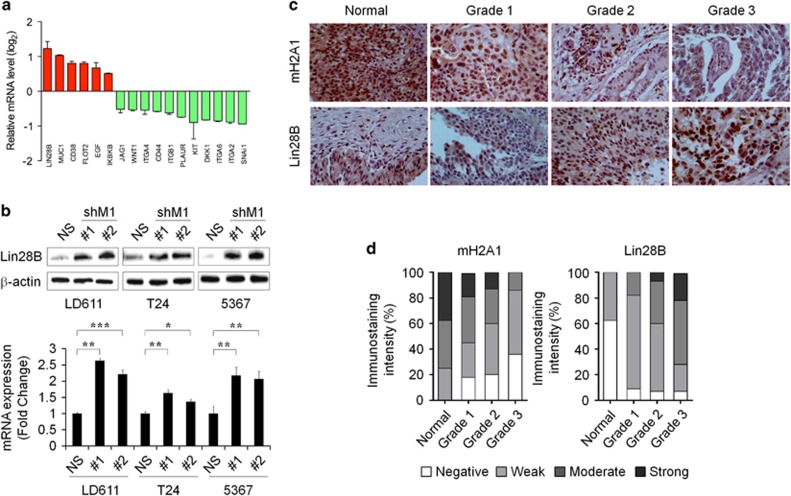
Lin28B is a downstream target of mH2A1. (**a**) We identified mH2A1 target genes in human cancer stem cell marker PCR arrays. (**b**) The expression of Lin28B in NS or shM1 bladder cancer cells was determined with western blot analysis and qPCR. (**c**) Bladder tumor tissue microarrays were evaluated with immunohistochemical analysis using an antibody against Lin28B. Original magnifications × 20 and × 40 are shown. (**d**) Immunostaining scores of macroH2A1 and Lin28B in bladder normal and malignant tissues. The graph indicates the percentage of sections with different scores (negative, weak, moderate and strong). *P*-values were calculated using the Student's *t*-test. *P<0.05, **P<0.01, ***P<0.001.

To identify mH2A1 target genes involved in induced stem-like properties, we performed a human cancer stem cell marker PCR array, which included 84 genes linked to cancer stem-like cells, in LD611 cells transfected with NS and shM1. The results indicate that Lin28B is the highest upregulated cancer stem cell factor by mH2A1 depletion in LD611 cells (Figure 2a; Supplementary Table S1). Moreover, in addition to Lin28B there are several putative cancer stem cell markers, such as *MUC1*, *CD38*, *FLOT2, EGF* and *IKBKB* that were also upregulated (signal log_2_ ratio>0.5) in mH2A1-depleted LD611 cells. Because Lin28B has been well characterized as a positive regulator of cancer progression and invasion in several types of cancers,^28, 29^ we selected Lin28B as the putative target gene of mH2A1 on the regulation of stem-like properties. The increased expression of Lin28B in mH2A1-depleted bladder cancer cell lines were validated by quantitative real-time PCR (qPCR) and western blot analysis (Figure 2b). We also confirmed that re-expression of mH2A1 in shM1 LD611 cells abolished mH2A1-depletion mediated Lin28B upregulation (Supplementary Figure S4a). Interestingly, we observed that mH2A1 expression was markedly lower in bladder cancer cell lines than in normal urothelial cells. In contrast, Lin28B expression was higher in bladder cancer cells than in normal cells (Supplementary Figure S4b). These results suggest that restricted expression of mH2A1 contributes to the stimulation of Lin28B expression in bladder cancer cells.

To investigate whether mH2A1 expression is inversely correlated with Lin28B expression in bladder cancer, we assessed the level of Lin28B expression in bladder-tissue microarrays containing 40 cases of bladder cancer tissues each at a different stage and grade of cancer and along with 8 cases of normal tissue. Representative images of immunohistochemical staining for mH2A1 and Lin28B are shown in Figure 2c. For mH2A1 staining, the majority of grade 3 bladder tumor tissuses (86%) was weak or negative, while the staining was moderate or strong in most normal (75%) tumor tissues. In contrast to the mH2A1 expression pattern, the highest expression of Lin28B was observed in grade 3 tumor tissues, while the lowest expression of Lin28B was observed in normal tissues (Figure 2d; Supplementary Table S2). These results indicate that lower levels of mH2A1 are associated with elevated Lin28B expression in malignant bladder tumor tissues. Taken together, the results demonstrated that Lin28B is a key target of mH2A1 and suggested that Lin28B induction by mH2A1 downregulation is responsible for enhanced stem-like properties.

### Loss of mH2A1 represses let-7 miRNA expression via Lin28B upregulation

**Figure 3:**
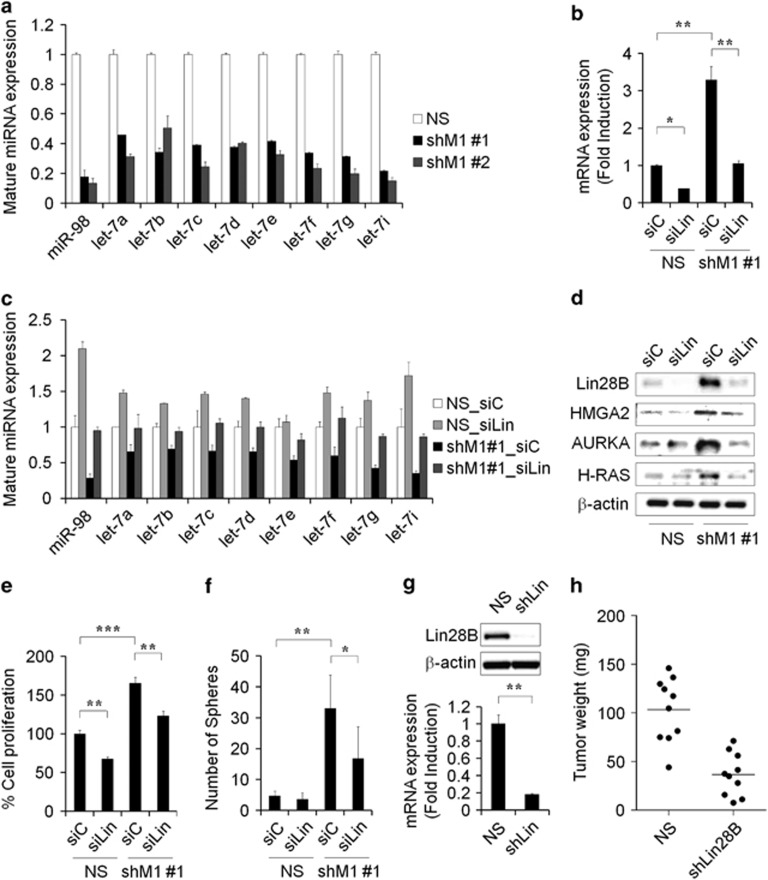
Loss of mH2A1 suppresses let-7 miRNA expression through Lin28B activation. (**a**) The levels of mature let-7 family miRNAs in NS or shM1 LD611 cells were evaluated with qPCR analysis. (**b**) siLin28B (siLin) or siControl (siC) RNA was transiently transfected into NS or shM1 LD611 cells. The efficiency of Lin28B knockdown was evaluated by qPCR. (**c**) The expression of mature let-7 family miRNAs was evaluated by qPCR. SNORD61 and SNORD68 were used as internal controls. (**d**) The expression of let-7 target proteins was analyzed by western blot analysis. (**e** and **f**) Control or Lin28B small interfering RNA was transiently transfected in NS or shM1 LD611 cells, and the effect of Lin28B knockdown on cell proliferation (**e**) and sphere formation (**f**) was determined. (**g**) The efficiency of Lin28B knockdown in LD611 cells was evaluated with western blot and qPCR. (**h**) NS or shLin28B LD611 cells were injected subcutaneously into nude mice (*n*=10). Forty days after injection, mice were killed and the tumor weight was measured. *P*-values were calculated using the Student's *t*-test. *P<0.05, **P<0.01, ***P<0.001.

As a knockdown of mH2A1 in LD611 cells led to the upregulation of Lin28B, we next investigated the level of mature let-7 miRNA expression in NS or shM1 LD611 cells. Lin28B is known as an inhibitor of the let-7 miRNA family and has been reported that repression of let-7 miRNAs augments breast cancer stem-like cell expansion.^15, 30^ In conjunction with this, let-7 target genes such as *HMGA2* and *AURKA* induce the self-renewal potential of hematopoietic stem cells and glioma-initiating cells, respectively.^31, 32^ The miRNA PCR array results showed that the let-7 family members were downregulated in shM1 cells when compared with expression in control cells (Supplementary Table S3). qPCR confirmed that mH2A1 knockdown reduced the expression of the let-7 miRNAs (Figure 3a). This suggests that the level of mH2A1 expression modulated let-7 miRNA by regulating Lin28B.

To further identify the role of Lin28B in let-7 miRNA regulation, we performed Lin28B loss of function studies by transfecting control and small interfering RNA against Lin28B into NS or shM1 LD611 cells and validating the knockdown efficiency of siLin28B with qPCR (Figure 3b). Our qPCR results showed that the knockdown of Lin28B restored the expression of mature let-7 miRNAs, which had been reduced by mH2A1 depletion in shM1-LD611 cells (Figure 3c). Next, we measured the levels of the let-7 target genes *HMGA2*, *AURKA* and *H-RAS*. The expression of these proteins was higher in mH2A1-depleted LD611 cells than in control cells (Figure 3d, lane 1 vs lane 3), and this induction was abolished by Lin28B depletion (Figure 3d, lane 3 vs lane 4). Induction of let-7 target gene expression by stable knockdown of mH2A1 may reflect an indirect effect, hence, we also determined whether mH2A1 small interfering RNA duplexes could alter the levels of Lin28B and let-7 target genes (Supplementary Figure S5). Next, we investigated whether Lin28B knockdown can rescue the cell proliferation and the tumor sphere formation. The results showed that the knockdown of Lin28B attenuated the proliferation and the sphere formation of LD611 cells induced by mH2A1 depletion (Figures 3e and f). In addition, mice injected with shLin28B LD611 cells had smaller sized tumors than those injected with control cells (Figures 3g and h).

**Figure 4:**
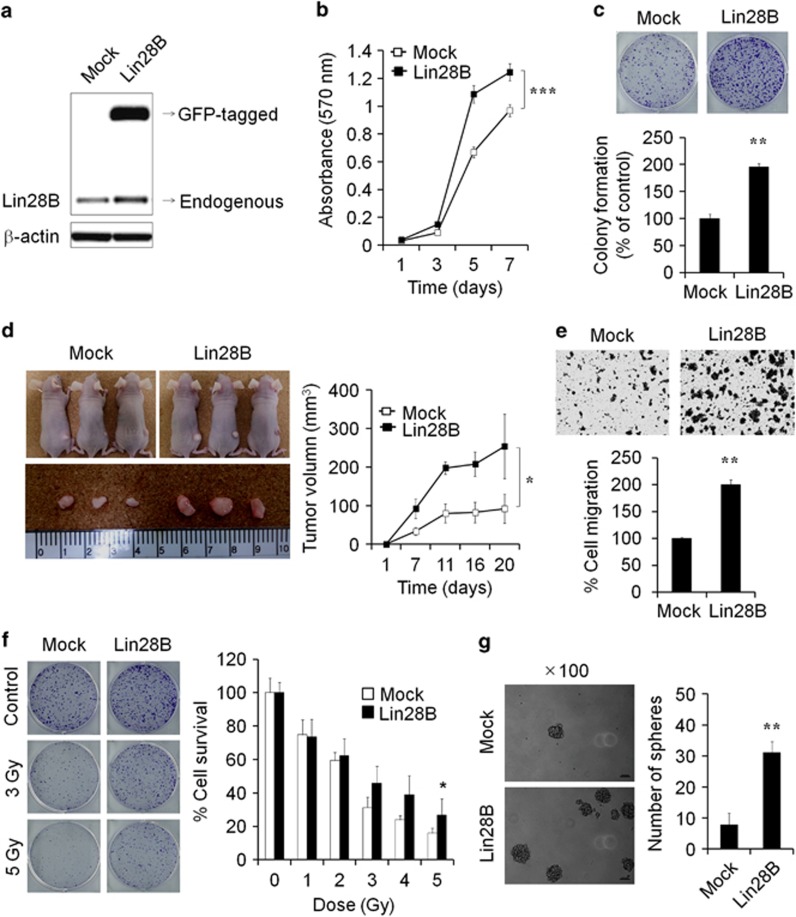
Activation of Lin28B correlates with induced tumorigenicity and increased migration. (**a**) LD611 cells were transfected with the pCMV6-AC-GFP or pCMV6-Lin28B-GFP vectors. To generate a stable cell line, cells were selected with geneticin (50 μg/ml). Expression of Lin28B was confirmed by western blot analysis. Mock: pCMV6 vector-expressing cells; Lin28B: pCMV6-Lin28B open reading frame vector-expressing cells. (**b** and **c**) The effect of Lin28B overexpression on bladder cancer cell proliferation was determined using MTT (**b**) and clonogenic (**c**) assays. (**d**) Mock or Lin28B LD611 cells were injected subcutaneously into nude mice (*n*=5). After 20 days, tumor mass was visualized (left), and the average tumor size was measured at different times (right). (**e**) The migration of Mock or Lin28B LD611 cells was evaluated. (**f**) Mock or Lin28B LD611 cells were left untreated or were treated with 1–5 Gy of IR, and cell survival rates were determined using the clonogenic assay. (**g**) Mock or Lin28B LD611 cells were incubated with DMEM/F12 supplemented with B27, N2, epidermal growth factor and basic fibroblast growth factor. After 1 week, spheres were counted and visualized. *P*-values were calculated using the Student's *t*-test. *P<0.05, **P<0.01, ***P<0.001.

We generated LD611 cells that were stably overexpressing Lin28B to examine the direct role of Lin28B in bladder cancer (Figure 4a). The results of the MTT and clonogenic assays indicated that overexpression of Lin28B significantly increased bladder cancer cell proliferation (Figures 4b and c). In conjunction with the results observed *in vitro*, tumor growth was faster in mice injected with Lin28B-overexpressing LD611 cells than in mice injected with the control cells (Figure 4d). To quantify this further, we examined whether overexpression of Lin28B promotes stem-like properties of LD611 cells. As expected, cell migration, radioresistance and sphere formation were significantly enhanced in Lin28B-overexprssing LD611 cells (Figures 4e–g). Collectively, these results indicated that the mH2A1/Lin28B/let-7 network has a crucial role in bladder cancer progression through modulating stem-like properties.

### The expression of Lin28B is regulated by mH2A1 occupancy on Lin28B promoter

**Figure 5:**
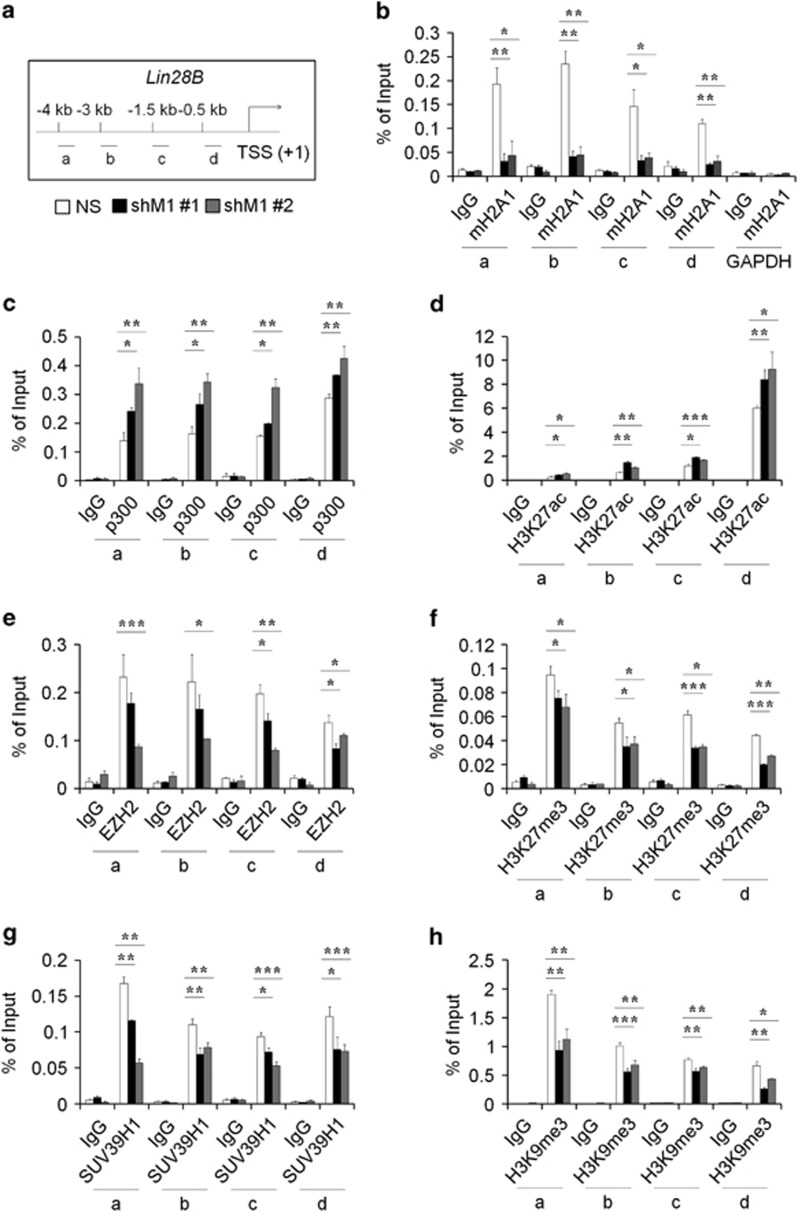
Reciprocal binding of p300 or EZH2/SUV39H1 regulates Lin28B expression. (**a**) Four regions at the *Lin28B* locus used in the ChIP assays. (**b**) ChIP assays were performed to evaluate the mH2A1 occupancy of the Lin28B promoter region in NS or shM1 LD611 cells. Chromatin was immunoprecipitated with a mH2A1 antibody. The results are shown as the percentage of the input chromatin (1%). GAPDH was used as an internal control; IgG was used as the antibody control. (**c**–**h**) ChIP experiments were performed using antibodies against p300 (**c**), H3K27ac (**d**), EZH2 (**e**), H3K27me3 (**f**), SUV39H1 (**g**) and H3K9me3 (**h**). *P*-values were calculated using the Student's *t*-test. *P<0.05, **P<0.01, ***P<0.001.

To determine whether mH2A1 directly regulates Lin28B expression, chromatin immunoprecipitation (ChIP) assays were performed. We probed four regions upstream from the transcription start site (Figure 5a). The results of the ChIP assays showed that mH2A1 occupancy in the promoter region of Lin28B was reduced in mH2A1-depleted bladder cancer cell lines (Figure 5b; Supplementary Figure S6). The occupancy of mH2A1 on GAPDH promoter was used as negative control. Because mH2A1 is known to interfere with the binding of chromatin remodeling factors thus leading to the condensed chromatin formation, we next investigated the levels of transcriptional coactivator (p300) or corepressors (EZH2 and SUV39H1) on the promoter of Lin28B in NS- or shM1-expressing LD611 cells. This showed that the depletion of mH2A1 significantly increased the occupancy of p300 on Lin28B promoter regions, and accordingly, acetylation of lysine 27 on histone H3 (H3K27ac) of the same region was enhanced in mH2A1-depleted LD611 cells compared with control cells (Figures 5c and d). Conversely, the localization of EZH2 (which methylates H3K27) on Lin28B promoter regions was restricted by mH2A1 depletion thereby decreasing the level of tri-methyl H3K27 (H3K27me3) at the promoter regions of the Lin28B locus (Figures 5e and f). We also determined the level of SUV39H1 (which methylates H3K9) at the Lin28B promoter region. Interestingly, the occupancy of SUV39H1 was also reduced by depletion of mH2A1 thus resulting in the decrease of H3K9me3 levels at the four upstream regions of the Lin28B locus (Figures 5g and f). The overall results demonstrate that the reciprocal bindings of coactivators and corepressors mediated by mH2A1 on the Lin28B promoter regulate the Lin28B expression and its downstream pathway.

## Discussion

Histone variants are implicated in multiple developmental processes, including the maintenance of pericentric heterochromatin, X-chromosome inactivation and germ cell differentiation.^33^ Histone variants are frequently deregulated in cancer cells, contributing to cancer initiation and progression.^5, 6, 34, 35, 36, 37^ Among the histone variants, mH2A has the most unique structure: it possesses a C-terminal non-histone domain of ~30 kDa.^38^ Recent studies have shown that the depletion of mH2A1 is able to enhance cancer progression and metastatic capacity by regulating oncogenes such as *CDK8*, c-*Fos* and *ERBB2*/*HER2*.^6, 38, 39^ Our *in vivo* experiments have shown that the knockdown of mH2A1 enhanced the tumorigenicity of LD611 bladder cancer cells (Figures 1a and c). These observations strongly support a tumor-suppressive role of mH2A1, which has been suggested in various types of cancers such as breast, melanoma and bladder cancers. In addition, several reports have shown that mH2A levels are elevated during embryonic stem cell differentiation in mice^8, 9^ and that mH2A acts as an epigenetic barrier during the reprogramming to induce pluripotency.^10, 11, 40^ Thus, the role of mH2A is not restricted to tumor suppression but also has a role in the stem cell regulation.

Cancer stem-like cells are unique cancer cells that have the characteristics of normal stem cells, and these cells can stimulate tumor growth through stem cell properties such as self-renewal and the ability to differentiate into various cell types.^41^ In addition, cancer stem-like cells are resistant to chemo- and radiotherapy. For example, CD133-expressing glioma cells are more resistant to radiation treatment than CD133-negative cell subpopulations and that the preferential checkpoint response in CD133-positive cells is responsible for this radioresistance.^22^ Consistent with this result, we found increased radioresistance and CHK2 phosphorylation specifically in mH2A1-depleted bladder cancer cells. It has also been suggested that lower levels of ROS are generated in breast cancer stem-like cells than in non-stem breast cancer cells, contributing to tumor radioresistance.^27, 42^ In this regard, reduced ROS levels in shM1 cells might be associated with an increase in the cancer stem-like cell subpopulation. Furthermore, we found that the loss of mH2A1 enhanced stem-like properties such as tumorigenicity and sphere-forming ability in bladder cancers (Figures 1c and d). We also showed that the SP fractions were expanded in shM1 LD611 cells compared with those in NS cells (Figure 1g), indicating that mH2A1 has a crucial role in induced stem-like properties.^26^

Lin28B was identified as a novel downstream target gene of mH2A1 from the human cancer stem cell marker PCR arrays, indicating that Lin28B is upregulated by mH2A1 depletion (Figure 2a). This upregulation of Lin28B has been found to increase the overall risk of mortality and relapse in cancer patients.^43^ A large body of evidence has also been suggested several oncogenic roles for Lin28B.^12, 44^ Our tissue microarray results supported this by revealing that Lin28B expression is upregulated in malignant bladder tissues when compared with normal tissues (Figure 2c). Because the induction of Lin28B inhibits the expression of mature let-7 miRNAs, we investigated the expression of mature miRNAs that are known to be involved in the cancer regulation after mH2A1 depletion. Importantly, almost all of the let-7 members were downregulated by more than twofold after mH2A1 depletion (Supplementary Table S1).^28, 43^ In addition, the reduced expression of let-7 miRNAs was completely rescued by knockdown of Lin28B. These observations suggest that the loss of mH2A1 is able to promote bladder cancer progression through the regulation of the Lin28B/let-7 axis. The tumor suppressor miRNA let-7, inhibits the expression of several oncogenes including *HMGA2* and *AURKA*.^45^ Both genes have been suggested to have essential roles in the regulation of self-renewal, differentiation and somatic cell reprogramming.^31, 46^ Hence, the Lin28B/let-7 regulatory circuit might also have an essential role in cancer stem-like cell regulation. Again, we found that enforced expression of Lin28B in LD611 cells enhanced the stem-like properties such as tumorigenicity, sphere-forming ability, migration capability and radioresistance (Figure 4). Moreover, ChIP assays showed that epigenetic modifiers such as p300, EZH2 and SUV39H1 have another important role in mH2A1-mediated Lin28B regulation (Figure 5). Collectively, these results provide evidence for the role that the mH2A1/Lin28B/let-7 regulatory circuit has in the development of bladder cancer stem cell-like properties.

**Figure 6:**
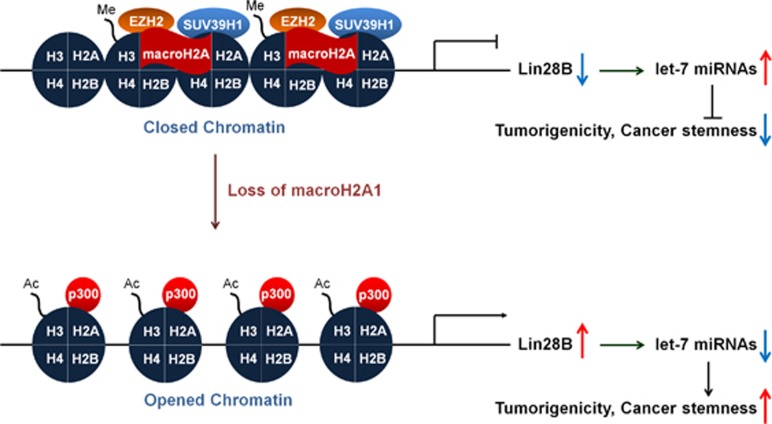
Model of the proposed regulatory mechanism involved in mH2A-mediated induction of tumorigenicity and cancer stemness. In the presence of mH2A, epigenetic repressors (EZH2/SUV39H1) are localized to the promoter of Lin28B. Accordingly, the repression of Lin28B enhances the mature let-7 miRNA production. On the other hand, the loss of mH2A upregulates Lin28B expression by recruitment of p300 to its promoter and Lin28B upregulation stimulates tumorigenicity and cancer stemness through the suppression of mature let-7 miRNA.

In conclusion, the results of this study show that a loss of mH2A1 contributes to the enhancement of bladder cancer tumorigenicity and stemness through regulating Lin28B/let-7 posttranscriptional gene regulatory network. In addition, we found that chromatin remodeling on Lin28B promoter regions by the reciprocal bindings of coactivators and corepressors mediated by mH2A1 regulates the Lin28B expression and its downstream pathway (Figure 6). Furthermore, our findings provide evidence for the role of mH2A1 in stem-like characteristics and demonstrate that novel downstream mechanisms are involved in bladder cancer progression and that more research needs to be accomplished to further quantify and identify pathways and regulators within this pathway that may lead to potential therapeutics in the treatment of bladder cancers.

## Materials and Methods

### Cell culture

Human bladder cancer cell lines (LD611, 5367 and T24) were grown at 37 °C under conditions of 20 O_2_ and 5% CO_2_ in Dulbecco's Modified Eagle's medium (DMEM; LD611), Roswell Park Memorial Institute medium (5367) or McCoy's 5 A (T24; WelGENE, Daegu-si, Korea) containing 10% fetal bovine serum (Hyclone, Logan, UT, USA) and 1% antibiotic-antimycotic solution (Gibco, Grand Island, NY, USA). Human urothelial cells (UROtsa) were grown in DMEM supplemented with 5% fetal bovine serum and 1% antibiotic-antimycotic solution.

### RNA interference and cell line generation

Non-target shRNA, shRNA specific for macroH2A1, and Lin28B in the lentiviral vector pLKO.1 were purchased from Sigma-Aldrich (St. Louis, MO, USA). Cells were transfected with shRNA constructs against macroH2A1 (#1; CTGAACCTTATTCACAGTGAA, #2; GCCAATGATGAAGAGCTGAAT), Lin28B (GCACCAGAAGAGCAAAGCAAA) or control along with psPAX2 and pMD2.G plasmid (Addgene, Cambridge, MA, USA) using Lipofectamine 2000 (Invitrogen, Carlsbad, CA, USA). Subsequently, the cells were infected with virus particles and the puromycin-selected cells were pooled. MacroH2A1 open reading frame in the pIRES-FLAG vector was transfected into the mH2A1-depleted LD611 cells to generate mH2A1 re-expressed cells. Lin28B-overexpressing stable cell line was generated by transfection with the control vector and Lin28B open reading frame in the pCMV6-AC-GFP vector (ORIGENE, Rockville, MD, USA) using Lipofectamine and selected with Geneticin. Small interfering RNA against macroH2A1 and Lin28B were purchased from Sigma-Aldrich.

### Migration assay

For the migration assay, LD611 cells (1 × 10^5^ cells/well) were seeded onto 8-μm transwell-inserts (Corning-Costar, Lowell, MA, USA). The lower chambers were filled with DMEM containing 10% fetal bovine serum. After 24 h, migrated cells were stained with crystal violet and counted using the Image-Pro Plus 7.0 software (Media Cybernetics, Rockville, MD, USA).

### *In vivo* experiments

Female BALB/c nude and SCID mice (4-weeks old) were obtained from Japan SLC, Inc., Shizuoka, Japan. All animal protocols used in this study were approved by the Institutional Animal Care and Use Committee at Dongnam Institute of Radiological & Medical Sciences (DIRAMS; Busan, Republic of Korea). SCID mice (*n*=10) were randomly divided into two groups and each mouse were transplanted with NS or shM1 LD611 cells and killed 30 days post injection. Total number of 32 nude mice were randomly divided into four groups (NS, shM1 #1, shM1 #2 and #1+M1; *n*=8 for each group) and each cell line (1 × 10^6^) was inoculated subcutaneously into nude mice. For testing the effect of Lin28B knockdown on tumor formation, nude mice (*n*=10 for each group) were injected with NS or shLin28B LD611 cells (1 × 10^6^). In case of Lin28B overexpression test, Mock or Lin28B-overexpressing LD611 cells (1 × 10^6^) were inoculated into nude mice (*n*=5 for each group). Tumor volume was estimated as follows: tumor volume=(short axis)^2^ × (long axis) × 0.5. All animal studies were followed by a blind randomized animal study protocol.

### Sphere-formation assay

Cells (1 × 10^3^ cells/well) were seeded on ultra-low attachment 24-well plates (Corning) in serum-free Dulbecco's modified Eagle medium/nutrient mixture F-12 (DMEM/F12; Invitrogen, Grand Island, NY, USA) supplemented with B27 (Invitrogen), N2 (Invitrogen), epidermal growth factor (20 ng/ml; Peprotech, London, UK) and basic fibroblast growth factor (10 ng/ml; Peprotech). After 1 week, spheres were visualized using a phase-contrast Nikon microscope (TS100; Tokyo, Japan).

### Western blot analysis

Western blot analysis was performed as described previously.^47^ The following primary antibodies were used: macroH2A1 (ab-37264, Abcam, Cambridge, MA, USA), Histone H3 (ab1791, Abcam), Histone H2A (Abcam), OCT4 (611203, BD Biosciences, San Jose, CA, USA), SOX2 (SC-20088, Santa Cruz, Santa Cruz, CA, USA), C-Myc (SC-40, Santa Cruz), KLF4 (ab75486, Abcam), HMGA2 (ab52039, Abcam), AURKA (610939, BD Biosciences), H-RAS (18295-1-ap, Proteintech, Chicago, IL, USA), Lin28B (#4196, Cell Signaling, Beverly, MA, USA), CHK1 (#2360, Cell Signaling), pCHK1 (#2348, Cell Signaling), CHK2 (#2662, Cell Signaling), pCHK2 (#2662, Cell Signaling) and β-actin (A5316, Sigma, St. Louis, MO, USA).

### Cell proliferation assay

Cell proliferation was assessed using the MTT and clonogenic assays. The MTT assay was performed as described previously.^47^ For the clonogenic assay, 5 × 10^3^ cells were seeded in a 6-well plate. After 2 weeks, colonies were stained with crystal violet and counted using the Image-Pro Plus 7.0 software (Media Cybernetics).

### IR treatment

Cells (1 × 10^3^ cells/well) were seeded in a six-well plate and incubated at 37 °C overnight. The next day, the cells were exposed to γ-rays from a ^137^Cs-ray source (Eckert & Ziegler, Berlin, Germany) at a dose rate of 2.6 Gy/min. After 2 weeks, the colonies were stained with crystal violet and counted using the Image-Pro Plus 7.0 software (Media Cybernetics).

### 2′,7′-dichlorofluorescein diacetate assay

Cells were harvested and washed with phosphate-buffered saline, and then treated with 2′,7′-dichlorofluorescein diacetate assay (10 μM). After 20 min, the 2′,7′-dichlorofluorescein diacetate assay-stained cells were washed with phosphate-buffered saline twice and analyzed using a FACSAria cell sorter (BD Biosciences).

### SP assay

SP assays were performed as described previously.^48^ Cells were incubated at 37 °C for 30 min with or without verapamil (150 μM; Sigma) and then stained with Hoechst 33342 (5 μg/ml; Invitrogen) for 90 min. After staining, the cells were treated with propidium iodide (2 μg/ml; BD Biosciences) to discriminate viable cells. Stained cells were analysed using a FACSAria cell sorter (BD Biosciences).

### Quantitative real-time PCR (qPCR)

qPCR was performed as described previously.^47^ For the miRNA PCR arrays, we purchased the miScript miRNA PCR Array kit (Human Cancer PathwayFinder) from QIAGEN (Valencia, CA, USA). Total RNA containing miRNA was extracted using an miRNeasy Mini Kit (QIAGEN), according to the manufacturer's protocol. To confirm let-7 miRNA expression, 1 μg of small RNA was reverse-transcribed using a miScript II RT kit (QIAGEN). The miScript universal primer (QIAGEN) was used as the antisense primer. SNORD61 and SNORD68 (QIAGEN) were used as the internal controls. The primers used for qPCR are listed in the Supplementary Table S4. For the cancer stem cell arrays, we used RT^2^ Profiler PCR Arrays (QIAGEN).

### Tissue microarrays and immunohistochemistry

Bladder tissue microarrays, containing 40 cases of bladder cancer tissues with different stages and grades of cancer and 8 cases of normal tissues, were purchased from US Biomax Inc. (Rockville, MD, USA). Paraffin sections were stained with macroH2A1 (ab-37264, Abcam) and Lin28B (ab-71415, Abcam) antibodies at 4 °C overnight. Then, the sections were incubated with secondary antibodies at room temperature for 1 h, followed by incubation with ABC solution (ABC reagent Elite Kit, Vector Laboratories, Burlingame, CA, USA) at room temperature for 1 h. After staining with diaminobenzidine, images were acquired using a Nikon ECLIPSE 80i microscope (Nikon). A scoring for immunohisochemical staining: negative or marginal (<20% of cells), weak (20–50% of cells), moderate (50–80% of cells) and strong (>80% of cells).

### Chromatin immunoprecipitation (ChIP)

ChIP assays were performed using a Magna ChIP kit (Millipore, Billerica, MA, USA) according to the manufacturer's protocol. The primers used for the ChIP assays are listed in the Supplementary Table S4. MacroH2A1 (07–219, Millipore), p300 (SC-584, Santa Cruz), EZH2 (ab3748, Abcam), SUV39H1 (ab12405, Abcam), H3K27ac (ab4729, Abcam), H3K27me3 (ab6002, Abcam), and H3K9me3 (ab8898, Abcam) antibodies were used to immunoprecipitate chromatin fragments.

### Statistical analysis

Each experiment was performed at least three times. All statistical analyses were performed using the Excel. The Student's *t*-test was used for statistical comparisons. *P<0.05, **P<0.01, ***P<0.001 were considered statistically significant.
