# Supplementary material for: MacroH2A1 downregulation enhances the stem-like properties of bladder cancer cells by transactivation of Lin28B
Source: Oncogene. 2015 Jun 1;35(10):1292–301. doi: 10.1038/onc.2015.187 (PMC4791524; doi:10.1038/onc.2015.187)
Supplement: Supplementary Table 1 [file onc2015187x1.doc]

**Table S1.** Analysis of cancer stem cell-related genes using RT^2^ Profiler PCR Arrays

| **Name** | **shM1/NS (log_2_)** |  | **Name** | **shM1/NS (log_2_)** |
| --- | --- | --- | --- | --- |
| LIN28B | 1.244 |  | PTPRC | -0.079 |
| MUC1 | 1.031 |  | KLF17 | -0.083 |
| CD38 | 0.807 |  | ATXN1 | -0.102 |
| FLOT2 | 0.803 |  | ALCAM | -0.112 |
| EGF | 0.680 |  | NOS2 | -0.120 |
| IKBKB | 0.509 |  | CD24 | -0.122 |
| YAP1 | 0.484 |  | SAV1 | -0.140 |
| NOTCH1 | 0.408 |  | MAML1 | -0.179 |
| BMI1 | 0.408 |  | LATS1 | -0.180 |
| NANOG | 0.394 |  | MYCN | -0.182 |
| POU5F1 | 0.373 |  | ALDH1A1 | -0.198 |
| FOXP1 | 0.322 |  | MERTK | -0.203 |
| DDR1 | 0.317 |  | ZEB2 | -0.206 |
| FZD7 | 0.277 |  | TGFBR1 | -0.242 |
| PTCH1 | 0.260 |  | STAT3 | -0.243 |
| HDAC1 | 0.242 |  | TWIST2 | -0.261 |
| MS4A1 | 0.214 |  | GATA3 | -0.269 |
| ZEB1 | 0.195 |  | ETFA | -0.301 |
| TWIST1 | 0.193 |  | FGFR2 | -0.303 |
| ERBB2 | 0.181 |  | PROM1 | -0.311 |
| NFKB1 | 0.179 |  | MYC | -0.319 |
| EPCAM | 0.153 |  | DLL1 | -0.346 |
| PLAT | 0.128 |  | AXL | -0.367 |
| ATM | 0.113 |  | DLL4 | -0.381 |
| TAZ | 0.108 |  | IL8 | -0.382 |
| LIN28A | 0.106 |  | GSK3B | -0.408 |
| WWC1 | 0.073 |  | PECAM1 | -0.430 |
| CHEK1 | 0.070 |  | FOXA2 | -0.439 |
| WEE1 | 0.049 |  | THY1 | -0.440 |
| NOTCH2 | 0.046 |  | ABCD5 | -0.449 |
| KITLG | 0.040 |  | CD34 | -0.498 |
| BMP7 | 0.037 |  | JAG1 | -0.514 |
| KLF4 | 0.036 |  | WNT1 | -0.533 |
| DACH1 | 0.032 |  | ITGA4 | -0.540 |
| JAK2 | 0.010 |  | CD44 | -0.578 |
| SMO | -0.001 |  | ITGB1 | -0.623 |
| SIRT1 | -0.006 |  | PLAUR | -0.744 |
| SOX2 | -0.017 |  | KIT | -0.824 |
| ENG | -0.023 |  | DKK1 | -0.826 |
| DNMT1 | -0.032 |  | ITGA6 | -0.854 |
| ID1 | -0.062 |  | ITGA2 | -0.884 |
| ABCG2 | -0.070 |  | SNAi1 | -0.942 |

**Table S2.** Analysis of mH2A1 and Lin28B expression using bladder cancer tissue arrays.

| **No.** | **Pathology diagnosis** | **Grade** | **mH2A1** | **Lin28B** |
| --- | --- | --- | --- | --- |
| 1 | Papillary transitional cell carcinoma | 1 | M | W |
| 2 | Papillary transitional cell carcinoma | 1 | W | W |
| 3 | Papillary transitional cell carcinoma | 1 | M | W |
| 4 | Papillary transitional cell carcinoma | 1 | S | M |
| 5 | Papillary transitional cell carcinoma | 1 | S | W |
| 6 | Transitional cell carcinoma | 1~2 | N | W |
| 7 | Papillary transitional cell carcinoma | 1 | W | W |
| 8 | Papillary transitional cell carcinoma | 1 | N | W |
| 9 | Papillary transitional cell carcinoma | 1~2 | W | W |
| 10 | Papillary transitional cell carcinoma | 1 | M | W |
| 11 | Papillary transitional cell carcinoma | 1 | N | W |
| 12 | Transitional cell carcinoma | 2 | M | M |
| 13 | Transitional cell carcinoma | 2 | S | M |
| 14 | Transitional cell carcinoma | 2 | W | W |
| 15 | Papillary transitional cell carcinoma | 1 | W | M |
| 16 | Papillary transitional cell carcinoma | 1~2 | M | W |
| 17 | Transitional cell carcinoma | 2 | M | W |
| 18 | Transitional cell carcinoma | 2 | W | N |
| 19 | Transitional cell carcinoma | 2 | W | M |
| 20 | Transitional cell carcinoma | 2 | N | W |
| 21 | Transitional cell carcinoma | 2 | W | W |
| 22 | Transitional cell carcinoma | 2 | N | W |
| 23 | Papillary transitional cell carcinoma | 1~2 | M | M |
| 24 | Papillary transitional cell carcinoma | 2 | W | M |
| 25 | Transitional cell carcinoma | 2~3 | W | N |
| 26 | Transitional cell carcinoma | 3 | M | W |
| 27 | Transitional cell carcinoma | 3 | W | M |
| 28 | Transitional cell carcinoma | 3 | N | M |
| 29 | Transitional cell carcinoma (sparse) | - | N | M |
| 30 | Transitional cell carcinoma | 3 | N | S |
| 31 | Transitional cell carcinoma | 3 | N | S |
| 32 | Transitional cell carcinoma | 3 | W | M |
| 33 | Transitional cell carcinoma | 3 | W | M |
| 34 | Transitional cell carcinoma | 3 | W | M |
| 35 | Transitional cell carcinoma | 3 | M | S |
| 36 | Transitional cell carcinoma | 3 | W | M |
| 37 | Transitional cell carcinoma | 3 | W | W |
| 38 | Transitional cell carcinoma | 2~3 | W | W |
| 39 | Squamous cell carcinoma | 2 | S | S |
| 40 | Mucinous adenocarcinoma | 1 | M | N |
| 41 | Normal urocystic tissue | - | S | N |
| 42 | Normal urocystic tissue | - | M | N |
| 43 | Normal urocystic tissue | - | W | N |
| 44 | Normal urocystic tissue | - | S | N |
| 45 | Normal urocystic tissue | - | W | N |
| 46 | Normal urocystic tissue | - | M | W |
| 47 | Normal urocystic tissue | - | M | W |
| 48 | Normal urocystic tissue | - | S | W |

N: Negative W: Weak M: Moderate S: Strong

**Table S3.** Identification of mH2A1 target miRNAs using miRNA PCR array

| **Name** | **shM1/NS (log_2_)** |  | **Name** | **shM1/NS (log_2_)** |
| --- | --- | --- | --- | --- |
| **hsa-miR-98-5p** | −2.169 |  | hsa-miR-30c-5p | −0.114 |
| **hsa-let-7d-5p** | −1.629 |  | hsa-miR-27b-3p | −0.108 |
| hsa-miR-96-5p | −1.535 |  | hsa-miR-23b-3p | −0.096 |
| hsa-miR-29b-3p | −1.515 |  | hsa-miR-181d | −0.018 |
| **hsa-let-7b-5p** | −1.490 |  | hsa-miR-19a-3p | −0.008 |
| hsa-miR-29a-3p | −1.408 |  | hsa-miR-15b-5p | −0.006 |
| hsa-miR-100-5p | −1.400 |  | hsa-miR-181a-5p | 0.013 |
| **hsa-let-7f-5p** | −1.363 |  | hsa-miR-125a-5p | 0.018 |
| hsa-miR-125b-5p | −1.356 |  | hsa-miR-128 | 0.038 |
| hsa-miR-155-5p | −1.232 |  | hsa-miR-335-5p | 0.041 |
| hsa-miR-222-3p | −1.117 |  | hsa-miR-20a-5p | 0.085 |
| hsa-miR-148b-3p | −1.041 |  | hsa-miR-140-5p | 0.118 |
| **hsa-let-7e-5p** | −1.019 |  | hsa-miR-146a-5p | 0.126 |
| **hsa-let-7g-5p** | −1.011 |  | hsa-miR-18a-5p | 0.144 |
| hsa-miR-130a-3p | −1.000 |  | hsa-miR-10b-5p | 0.194 |
| hsa-miR-210 | −0.981 |  | hsa-miR-200c-3p | 0.234 |
| hsa-miR-181c-5p | −0.979 |  | hsa-miR-149-5p | 0.295 |
| **hsa-let-7i-5p** | −0.973 |  | hsa-miR-20b-5p | 0.303 |
| **hsa-let-7a** | −0.935 |  | hsa-miR-146b-5p | 0.375 |
| hsa-miR-203a | −0.820 |  | hsa-miR-34a-5p | 0.416 |
| hsa-miR-301a-3p | −0.812 |  | hsa-miR-34c-5p | 0.416 |
| hsa-miR-378a-3p | −0.780 |  | hsa-miR-9-5p | 0.472 |
| hsa-miR-148a-3p | −0.761 |  | hsa-miR-92a-3p | 0.680 |
| hsa-miR-17-5p | −0.759 |  | hsa-miR-133b | N.D. |
| hsa-miR-126-3p | −0.723 |  | hsa-miR-122-5p | N.D. |
| hsa-miR-132-3p | −0.706 |  | hsa-miR-184 | N.D. |
| hsa-miR-181b-5p | −0.685 |  | hsa-miR-214-3p | N.D. |
| hsa-miR-205-5p | −0.659 |  | hsa-miR-373-3p | N.D. |
| hsa-miR-32-5p | −0.643 |  | hsa-miR-206 | N.D. |
| hsa-miR-193b-3p | −0.630 |  | hsa-miR-124-3p | N.D. |
| hsa-miR-21-5p | −0.589 |  | hsa-miR-1 | N.D. |
| hsa-miR-218-5p | −0.571 |  | hsa-miR-150-5p | N.D. |
| hsa-miR-191-5p | −0.568 |  | hsa-miR-127-5p | N.D. |
| hsa-miR-7-5p | −0.559 |  | hsa-miR-144-3p | N.D. |
| hsa-miR-10a-5p | −0.490 |  | hsa-miR-143-3p | N.D. |
| hsa-miR-25-3p | −0.432 |  | hsa-miR-215 | N.D. |
| hsa-miR-135b-5p | −0.397 |  | hsa-miR-372 | N.D. |
| hsa-miR-16-5p | −0.368 |  | hsa-miR-39-3p | N.D. |
| hsa-miR-183-5p | −0.347 |  | hsa-miR-193a-5p | N.D. |
| hsa-miR-196a-5p | −0.311 |  | hsa-miR-142-5p | N.D. |
| **hsa-let-7c** | −0.269 |  | hsa-miR-138-5p | N.D. |
| hsa-miR-15a-5p | −0.140 |  | hsa-miR-134 | N.D. |
| hsa-miR-27a-3p | −0.136 |  |  |  |

N.D.: Not determined Gray box: let-7 family miRNAs

**Table S4.** Primers used in this study

qRT-PCR primers

| Gene | Forward (5’-3’) | R (5’-3’) |
| --- | --- | --- |
| MacroH2A1 | ACA ACCGAGGGCACACCT | CAAAGCCGGCTAAATTACTGA |
| Lin28B | AGCCCCTTGGATATTCCAGTC | AATGTGAATTCCACTGGTTCTCCT |
| β-actin | AGCGAGCATCCCCCAAAGTT | GGGCACGAAGGCTCATCATT |

ChIP primers

| Gene | Forward (5’-3’) | R (5’-3’) |
| --- | --- | --- |
| Lin28B (-0.5) | TATTTAAACGACCCCCTCCC | AACACAATGGAGGGACCTTG |
| Lin28B (-1.5) | CTCGGTAGCTTTGTCGAAGG | AAATGCACCACCAGACTTGC |
| Lin28B (-3) | TGCAGGAATAAAACACCCCT | TCACAGAAAGTCGTCCTATGAGT |
| Lin28B (-4) | GCAGAGAGAGATCCCGAAAA | TCCGAACCCAAGAAAATGAG |

miRNA primers

| Name | Sense | Antisense |
| --- | --- | --- |
| hsa-miR-98-5p | TGAGGTAGTAAGTTGTATTGTT | miScript Universal Primer |
| hsa-let-7a-5p | TGAGGTAGTAGGTTGTATAGTT |  |
| hsa-let-7b-5p | TGAGGTAGTAGGTTGTGTGGTT |  |
| hsa-let-7c-5p | TGAGGTAGTAGGTTGTATGGTT |  |
| hsa-let-7d-5p | AGAGGTAGTAGGTTGCATAGTT |  |
| hsa-let-7e-5p | TGAGGTAGGAGGTTGTATAGTT |  |
| hsa-let-7f-5p | TGAGGTAGTAGATTGTATAGTT |  |
| hsa-let-7g-5p | TGAGGTAGTAGTTTGTACAGTT |  |
| hsa-let-7i-5p | TGAGGTAGTAGTTTGTGCTGTT |  |
